# Mapping hippocampal glutamate in healthy aging with *in vivo* glutamate-weighted CEST (GluCEST) imaging

**DOI:** 10.3389/fnagi.2024.1535158

**Published:** 2025-01-24

**Authors:** Maggie K. Pecsok, Heather Robinson, Ally Atkins, Monica E. Calkins, Mark A. Elliott, Arianna Mordy, Jacquelyn Stifelman, Ruben C. Gur, Paul J. Moberg, Ravi Prakash Reddy Nanga, Kosha Ruparel, Russell T. Shinohara, David A. Wolk, Ravinder Reddy, David R. Roalf

**Affiliations:** ^1^Brain and Behavior Lab, Neurodevelopment and Psychosis Section, Department of Psychiatry, University of Pennsylvania Perelman School of Medicine, Philadelphia, PA, United States; ^2^Department of Psychological Sciences, Institute for the Brain and Cognitive Sciences, University of Connecticut, Storrs, CT, United States; ^3^Department of Child and Adolescent Psychiatry and Behavioral Sciences, Lifespan Brain Institute (LiBI) of CHOP and Penn Medicine, Children’s Hospital of Philadelphia, Philadelphia, PA, United States; ^4^Center for Advanced Metabolic Imaging in Precision Medicine (CAMIPM), Perelman School of Medicine, University of Pennsylvania, Philadelphia, PA, United States; ^5^Cognitive and Clinical Neuroscience Lab, UCLA Brain Mapping Center, Department of Psychiatry and Behavioral Sciences, University of California, Los Angeles, Los Angeles, CA, United States; ^6^Penn Statistics in Imaging and Visualization Endeavor (PennSIVE), Perelman School of Medicine, University of Pennsylvania, Philadelphia, PA, United States; ^7^Penn Memory Center and Alzheimer's Disease Research Center, Perelman School of Medicine, University of Pennsylvania, Philadelphia, PA, United States

**Keywords:** glutamate, aging, 7Tesla MRI, GluCEST, hippocampus

## Abstract

**Introduction:**

Hippocampal glutamate (Glu) dysfunction is a pertinent indicator of neurodegeneration, yet mapping typical age-related changes in Glu has been challenging. Here, we use a 7T MRI approach, Glutamate Chemical Exchange Saturation Transfer (GluCEST), to measure bilateral hippocampal Glu in healthy old (HOA) and young (HYA) adults.

**Methods:**

Bilateral hippocampal GluCEST data was acquired from 27 HOA and 22 HYA using 7T MRI. GluCEST differences by age and hemisphere were tested with a linear mixed model. GluCEST asymmetry index was also evaluated by age. Exploratory analyses examined associations between hippocampal GluCEST, age group, and scores on the Montreal Cognitive Assessment (MoCA) and Cognitive Complaints Index (CCI).

**Results:**

GluCEST levels showed an age group and hemisphere interaction. In HOA, GluCEST was higher in left than right hippocampus, but in HYA, GluCEST level was equivalent across hemispheres. HOA had lower GluCEST than HYA in the right hippocampus. GluCEST asymmetry index confirmed significant left asymmetry in HOA. Lower GluCEST levels in HOA were associated with subjective cognitive complaints as measured by the CCI.

**Discussion:**

Hippocampal GluCEST provides insight into age-related neural changes, with lower GluCEST in the right hippocampus in older adults. These findings offer a step toward elucidating the asymmetrical trajectory of hippocampal glutamatergic alterations and their relationship to cognitive phenotypes.

## Introduction

Among the biological mechanisms underlying age-related neuronal and synaptic changes, altered brain glutamate (Glu)—the most abundant excitatory neurotransmitter in the mammalian brain—is considered critical given its ubiquitous role in brain function ranging from cellular energetics to high-order cognitive functions (Coyle, 2006; Curtis and Johnston, 1974; Erecińska and Silver, 1990; McEntee and Crook, 1993; Roalf et al., 2020; Robbins and Murphy, 2006). Age-related disruptions in Glu signaling, particularly in the hippocampus, have been linked to the degradation of neural communication and information processing efficiency. For example, there is evidence demonstrating that alterations in NMDA-mediated Glu neurotransmission in the aging hippocampus—driven by disrupted calcium homeostasis, changes in subunit expression, and other mechanisms—impairs long-term potentiation (LTP) and contributes to cognitive decline (Kumar et al., 2019). Similarly, cognitive deficits in aged rats are associated with a reduced surface expression of GluR1-containing AMPA receptors in the hippocampus, which are essential for Glu-mediated synaptic plasticity (Yang et al., 2015). Finally, alterations in Glu transmission and homeostasis are exacerbated in neurodegenerative diseases, highlighting the importance of understanding Glu alterations in the aging human brain (Castillo-Vazquez et al., 2024). Indeed, recent advances in neuroimaging have led to a better understanding of neurometabolic dysfunction in aging and may ultimately enable earlier detection and targeted treatments for age-related disorders. In humans, the most comprehensive data on the neurometabolic profile of the brain is from post-mortem tissue (Hynd et al., 2003). More recently, high-field, non-invasive proton magnetic resonance spectroscopy (^1^HMRS) has enabled the *in vivo* study of neurometabolites in the human brain (Soares and Law, 2009), including in aging (Cleeland et al., 2019).

To date, ^1^HMRS has been the primary method used for *in vivo* Glu/Glx imaging in aging. As we reported in a meta-analysis of ^1^HMRS studies (Roalf et al., 2020), Glu deficits in healthy aging are moderate-to-large but with significant heterogeneity across studies. ^1^HMRS at 3 Tesla (3T) MRI allows for high-quality measurement of many brain metabolites across brain regions (e.g., Barker et al., 2001). Yet, the quantification of Glu remains particularly challenging at 3T MRI due to the overlap of signal from Glu and glutamine (Gln) resonances (Ramadan et al., 2013). Given these challenges, ^1^HMRS data are often collected across large volumes of interest and the signals from Glu and Gln are often combined (Glx) to improve signal-to-noise ratio. Thus, the ability to specifically elucidate Glu levels in age-relevant brain structures, most importantly the hippocampus, has been challenging.

Advances in ultra-high field 7T MRI and the application of novel imaging techniques tailored to probe Glu, such as Glutamate-weighted Chemical Exchange Saturation Transfer (GluCEST), offer a unique opportunity to investigate brain Glu more precisely. GluCEST produces images weighted for Glu (Cai et al., 2012) with an unrivaled spatial resolution advantage compared to ^1^HMRS (Cember et al., 2023). The high degree of intra-day and inter-day reliability of this method was also shown in healthy volunteers at 7T (Nanga et al., 2018). Furthermore, human clinical applications have demonstrated that GluCEST is a powerful tool for probing neurological and psychiatric conditions where other forms of non-invasive physiological measurement are limited. GluCEST studies have produced advances in epilepsy (Davis et al., 2015), schizophrenia and related psychoses (Debnath et al., 2020; Roalf et al., 2017; Sydnor et al., 2021), hyperinsulinism/hyperammonemia (HI/HA) syndrome (Rosenfeld et al., 2022) and multiple sclerosis research (O’Grady et al., 2019). However, there has been no assessment of GluCEST in healthy older adults. Thus, a refined non-invasive measurement of brain Glu using GluCEST can improve our understanding of Glu in aging and contribute to identifying potential targets for intervention and prevention of age-related disorders.

The hippocampus is essential for learning and memory and declines in volume with age (Lister and Barnes, 2009; Nobis et al., 2019). Large-scale studies suggest that age-related declines in gray matter volume, including within medial temporal lobe regions, may not be symmetric across hemispheres (Dolcos et al., 2002; Minkova et al., 2017; Nobis et al., 2019). The neurobiological mechanism for hippocampal volume decline remains unclear, but it is likely that neurometabolic changes, including changes in brain Glu, play a role (Cox et al., 2022). Glutamatergic neurotransmission is vital for long-term potentiation and other hippocampal processes that support learning and memory. Notably, the left and right hippocampi have distinct properties, ranging from gene expression (Jordan, 2020; Klur et al., 2009; Moskal et al., 2006) to glutamatergic signaling patterns (Shinohara and Hirase, 2009). Aging affects hippocampal Glu signaling and metabolism through changes in receptor function and increased neuroinflammation, and these changes likely contribute to age-related cognitive decline (Kumar et al., 2019; Ménard and Quirion, 2012; Rozycka et al., 2019; Song et al., 2021; Roalf et al., 2020). Given previous findings of asymmetrical volume loss and the central role of Glu in hippocampal functioning, we measured and compared bilateral hippocampal brain Glu in healthy older and young adults using 7T GluCEST MRI. We hypothesized that Glu levels would be lower in healthy older adults.

## Methods

### Participants

Twenty-seven (*n* = 27) healthy older adults [HOA; age: 72 (±4); 74% Female] and 22 young healthy adults [HYA; age: 22 (±4); 55% Female] were imaged using 7T MRI (Table 1). Older adults were recruited from the Penn Memory Center and the Clinical Core of the University of Pennsylvania’s Alzheimer’s Disease Research Center between August 2020 and July 2022. For older adults, comprehensive diagnostic assessments were conducted by experienced clinicians, including review of medical history, neuroimaging, psychometric, and laboratory data, as well as physical, psychiatric, and neurologic examinations (Roalf et al., 2012). Based on these data, participants with dementia and other neurological or psychiatric conditions were excluded. Young adults were recruited from the Brain Behavior Laboratory and Lifespan Brain Institute in the Department of Psychiatry at the University of Pennsylvania. Young adults underwent diagnostic assessments that included self-reported neurological history and psychiatric assessment (Calkins et al., 2017). To ensure our sample was optimized for studying the effects of healthy aging, older and young adults with a neurological or psychiatric disorder or any contraindication to 7T MRI were excluded. All participants provided written informed consent prior to study participation.

**Table 1:** Summarizes demographics data and cognitive testing scores in healthy older adults and healthy younger adults.

	HOA (*N* = 27)	HYA (*N* = 22)	*p*-value
Age			
Mean (SD)	71.6 (4.23)	21.7 (3.76)	<0.001
Median [Min, Max]	71.0 [66.0, 80.0]	20.5 [18.0, 30.0]	
Sex			
Female	20 (74.1%)	12 (54.5%)	0.26
Male	7 (25.9%)	10 (45.5%)	
Race			
Black or African American	6 (22.2%)	4 (18.2%)	<0.001
Other*	0 (0%)	10 (45.5%)	
White	21 (77.8%)	8 (36.4%)	
MoCA			
Mean (SD)	27.4 (3.09)	26.9 (3.39)	0.571
Median [Min, Max]	29.0 [16.0, 30.0]	28.0 [20.0, 30.0]	
CCI**			
Mean (SD)	42.2 (19.3)	22.6 (5.61)	0.0109
Median [Min, Max]	41.5 [20.0, 88.0]	20.0 [20.0, 41.0]	

^*^Asian (*n* = 6); Hawaiian/Pacific Islander (*n* = 1); More than one race (*n* = 3). ^**^CCI data was available for a subset of participants (*n* = 10 HOA, 22 HYA).

### 7T structural and GluCEST MRI acquisition

**Figure 1:**
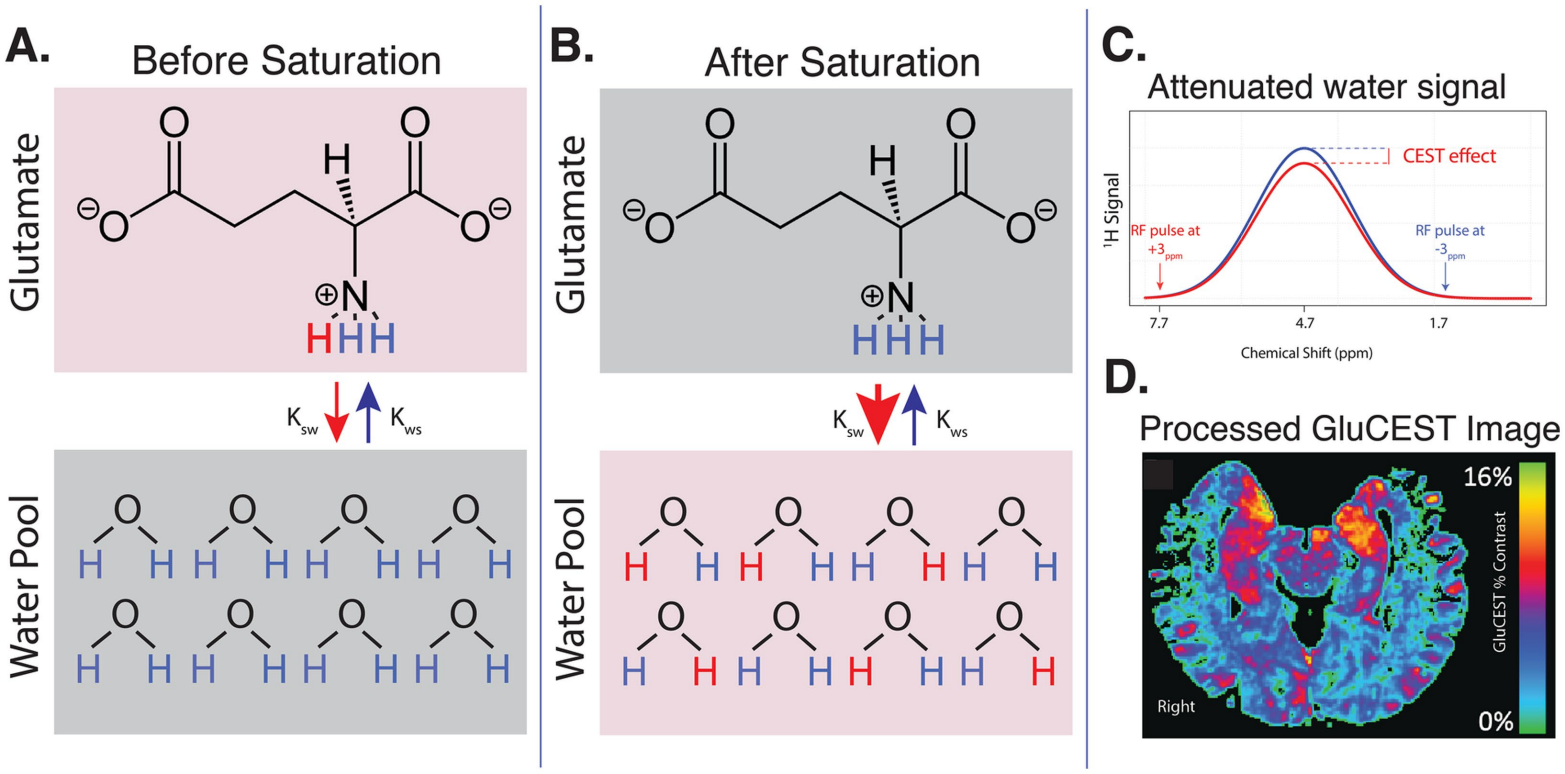
Summary of GluCEST technique. **(A)** Before saturation, an RF coil is used to selectively saturate Glu protons at a frequency of +3 ppm relative to water. These saturated glutamate protons exchange with protons in the bulk water pool, allowing the saturation to transfer to water molecules. **(B)** After saturation, many saturated Glu protons have exchanged with protons from the water pool. Control saturation pulses at −3 ppm are applied to account for the effects of macromolecules and magnetization transfer, isolating the specific contribution of Glu. **(C)** The difference in the water signal before and after saturation with +3 ppm versus −3 ppm pulse is measured and quantified as the GluCEST effect. This represents the change in water signal due to proton exchange with glutamate. **(D)** After correcting for field inhomogeneities and tissue water density, the processed GluCEST image now captures regional Glu concentrations.

MRI data for all main analyses were acquired on a 7T Siemens Terra scanner at the University of Pennsylvania with a single-channel transmit and 32-channel receive head coil (Nova Medical, Wilmington, MA, USA). Structural images including T_1_-weighted uniform (UNI) images and corresponding inversion (INV1 and INV2) images were acquired with a Magnetization Prepared 2 Rapid Acquisition Gradient Echoes (MP2RAGE) sequence (Marques et al., 2010) with the following parameters: echo time (TE) = 2.94 ms, repetition time (TR) = 4,900 ms, inversion time (TI) = 700/2700 ms, 192 sagittal slices, resolution = 0.9×0.9×0.9mm^3^, matrix = 256×256, FOV = 230. 2D GluCEST (slice number: 1, slice thickness: 5 mm) data was acquired in an axial slice positioned through bilateral hippocampi and positioned to maximize coverage of hippocampal structures in the 2D acquisition plane (Figure 1). Acquisition of GluCEST images and B_0_ and B_1_ maps was performed. GluCEST imaging, previously described in greater detail (Cai et al., 2012; Cember et al., 2023; Jacobs et al., 2022; Kogan et al., 2013), is summarized in Figure 1. Briefly, the RF coil selectively saturates glutamate (Glu) protons at +3 ppm. Saturated protons then exchange with water protons, thereby attenuating the local water signal (Figure 1B). Control pulses at −3 ppm do not saturate Glu protons and are used to account for macromolecule effects. The GluCEST effect then is calculated as the difference in water signal after +3 ppm versus −3 ppm pulses. After correcting for field inhomogeneities and water density, the processed GluCEST image captures local Glu concentrations with high spatial precision (Figure 1D). The GluCEST field of view used the in the present study offered full in-plane brain coverage (matrix size: 224 × 224) and 1 × 1 mm^2^ in-plane resolution (Cai et al., 2012; Calkins et al., 2017). All data passed quality assurance for gross motion or inhomogeneity artifacts. Automated measures of head motion (mean and maximum head displacement) were estimated using previously reported methods (Roalf et al., 2016; Satterthwaite et al., 2012) and are reported in the Supplementary material. A subset of participants (*n* = 14 HYA, *n* = 26 HOA) underwent 3T T_1_w MRI (see acquisition details in Supplementary material). These data were used for sensitivity analyses as described below.

### MRI data processing

7T MP2RAGE INV2 images were used to register anatomical regions of interest (i.e., hippocampus) to the GluCEST acquisition. INV2 images were corrected for field bias using Advanced Normalization Tools (ANTs) N4 (Avants et al., 2011) and used for tissue segmentation and atlas registration. FSL FAST (Zhang et al., 2001) was employed to generate three tissue segmentation maps and gray matter density maps. To register atlases from MNI space to participant images, INV2 images were first non-linearly registered to the MNI152 nonlinear T_1_w template using ANTs symmetric diffeomorphic image normalization (Avants et al., 2011).

**Figure 2:**
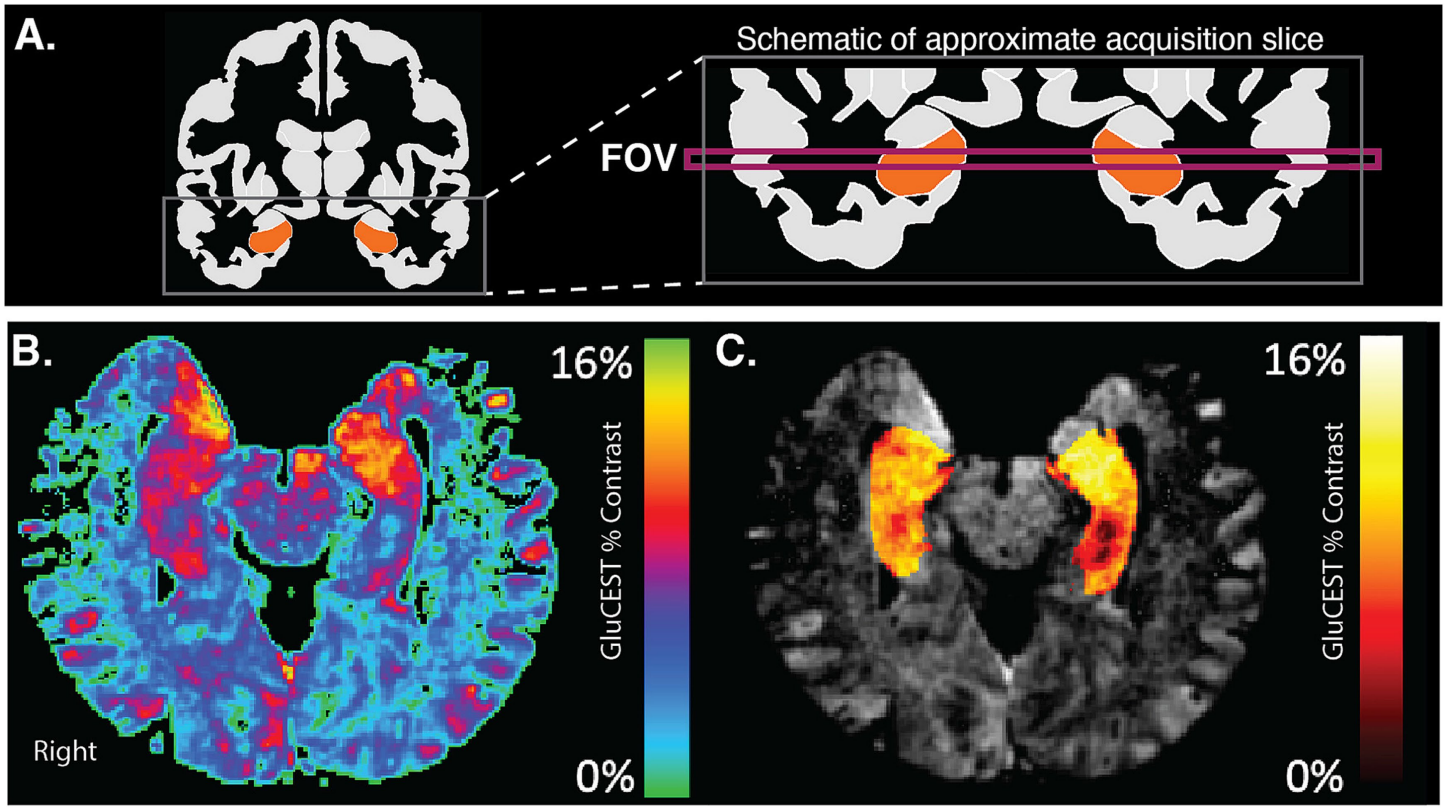
GluCEST acquisition in hippocampus. **(A)** The schematic shows a coronal view of the acquired FOV for GluCEST acquisition. This FOV captures portions of left and right hippocampi. **(B)** An example GluCEST map in the axial plane. Areas of the hippocampus show high levels (red-yellow) of GluCEST signal. **(C)** GluCEST map after masking with hippocampal region-of-interest and overlaying on an anatomic image.

GluCEST images represent the GluCEST contrast in each voxel (in arbitrary units). GluCEST images were first corrected for B_0_ and B_1_ inhomogeneity effects using Python software (pyGluCEST (Roalf et al., 2022)), as previously described (Cai et al., 2012; Roalf et al., 2017; Sydnor et al., 2021). Voxels that had a B_0_ offset of greater than ±1 ppm and voxels with relative B_1_ values outside of the 0.3–1.3 range were excluded (Roalf et al., 2017), as were voxels labeled as cerebrospinal fluid by tissue segmentation maps. GluCEST values were independently extracted from the left and right hippocampus (Figure 2C). Hippocampal volume covered by the 2D GluCEST scan was calculated for each participant. Scanning quality was high in all participants; mean and maximum head displacement during scanning were low for all participants and did not differ between age groups (see Supplementary Table S1).

### Cognitive screening

All participants were screened for global cognitive impairment using the Montreal Cognitive Assessment (MoCA) (Nasreddine et al., 2005). Subjective rating of cognitive functioning was assessed in a subset of participants (*n* = 22 HYA, *n* = 10 HOA) using the Cognitive Complaints Index (CCI) (Wang et al., 2006), a 20-item assessment on which higher scores indicate greater subjective cognitive decline. We note here that COVID-19 restrictions during the study limited the length of study visits for some participants, resulting in incomplete behavioral data in some cases. Because of these restrictions, only 10 HOA participants were able to complete the CCI during their in-person visit. No CCI data were specifically excluded, and no participants were selectively prevented from completing the assessment.

### Statistical analysis

Data were compared across groups using *t*-tests and chi-square where appropriate. GluCEST age effects were tested with a linear mixed model using GluCEST level as the dependent variable, age group (young, old), hemisphere (right, left) as the fixed effects, and participant as the random effect. Sex and 2D hippocampus volume were included as covariates. Post-hoc comparisons were completed using least squares means approach (‘lsmeans’) (Lenth, 2016). Asymmetry index was calculated in line with previous studies (Sarica et al., 2018) as the percent difference in left versus right GluCEST levels: (Left – Right)/((Left + Right)/2)*100. Asymmetry was compared across groups using a linear model with GluCEST asymmetry as the dependent measure, age group (young, old) as the independent predictor, and sex and 2D volume of GluCEST as covariates. Sensitivity analyses for the above models leveraged the 3T T_1_w images to incorporate 3D hippocampal volume, rather than 2D GluCEST acquisition volume, as a covariate. The association between GluCEST levels and cognitive screening measures (MoCA, CCI) was assessed using an exploratory linear model. Specifically, the model examined the interaction between GluCEST levels, hemisphere, and age group in predicting cognitive scores. For all analyses, significance values were set at alpha = 0.05 and the Benjamini-Hochberg method was used to correct for family-wise multiple comparisons. All *p*-values reported are false discovery rate (FDR) corrected. A post-hoc power analysis was also conducted (see Supplementary material). All statistics were computed in R (v4.3.1).

## Results

### GluCEST in left and right hippocampus across age groups

**Figure 3:**
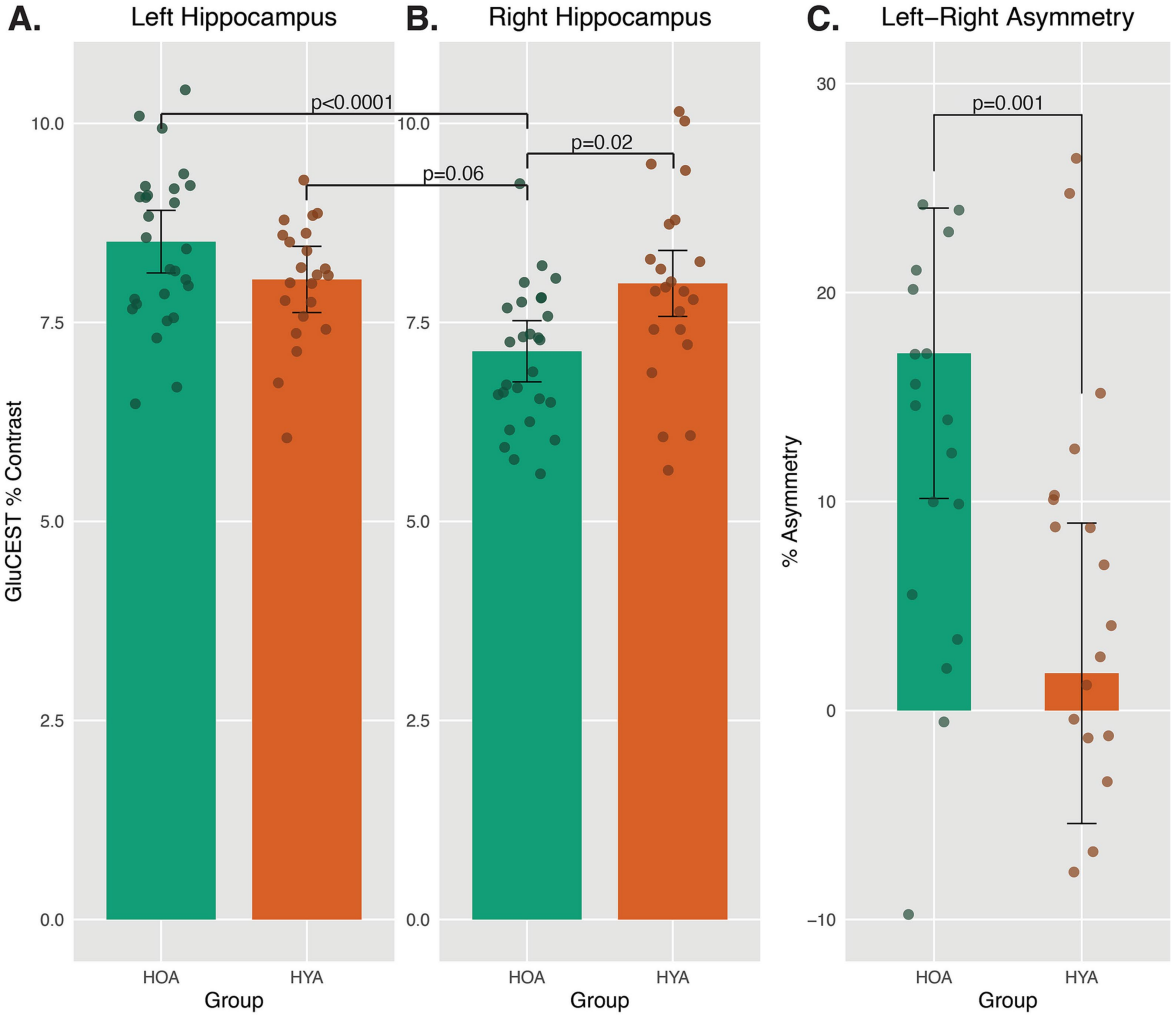
Asymmetric GluCEST levels in HOA and HYA. **(A,B)** Average GluCEST levels in Left and Right Hippocampus for HOA and HYA are presented. A mixed model revealed an interaction between age group and hemisphere on GluCEST level. Pairwise comparison showed no group difference in Left hippocampus GluCEST levels and no hemispheric difference in GluCEST levels in HYA. Right hippocampus GluCEST was lower in HOA as compared to HYA. In HOA, Right hippocampus GluCEST was lower than Left hippocampus GluCEST. Least square means ± 95% CI. **(C)** Percent asymmetry was calculated as ((Left–Right hippocampus GluCEST)/(Left+Right hippocampus GluCEST))*100, per previous studies (Sarica et al., 2018). HOA had significantly higher leftward asymmetry as compared to HYA. Positive numbers indicate more leftward asymmetry.

The mixed model analysis on GluCEST levels yielded a significant interaction between age group and hemisphere, *F*(1, 47.1) = 12.89, p_FDR_ = 0.006. Post-hoc pairwise comparison showed that, among HOA, GluCEST was higher in the left as compared to the right hippocampus, *t*(47.6) = 5.42, p_FDR_ < 0.0001, Cohen’s d effect size = 1.60 (Figures 3A,B, green bars). However, among HYA, there was no significant lateralized difference in hippocampal GluCEST (Figures 3A,B, orange bars). In the right hippocampus, HOA had lower GluCEST than HYA, *t*(89.6) = 2.99, p_FDR_ = 0.02, Cohen’s d effect size = 0.63 (Figure 3B). Right hippocampus GluCEST level in HOA was also nominally lower than left hemisphere GluCEST level in HYA, *t*(89.6) = 3.16, p_FDR_ = 0.06, Cohen’s d effect size = 0.67. The above results included 2D acquisition volume and sex assigned at birth, neither of which differed across groups (p_FDR_ > 0.05), as covariates. As expected, HOA had lower 3D volume of the left (*t*(22.3) = −4.03, p_FDR_ = 0.003) and right [*t*(25.3) = −3.45, p_FDR_ = 0.005] hippocampus as compared to HYA when measured using high resolution 3T MRI. Sensitivity analyses using 3T hippocampal volume instead of 7T 2D slice volume did not change the GluCEST results reported above (see Supplementary material). Sensitivity analysis was also conducted to stratify results by biological sex, showing similar effects as above in both for men and women (see Supplementary material).

### Asymmetry index of hippocampal GluCEST

HOA had larger left asymmetry in GluCEST than HYA, *F*(1,45) = 13.07, p_FDR_ = 0.025, Cohen’s d effect size = 1.06 (Figure 3C). This analysis included 2D volume as a covariate, and results were similar in sensitivity analysis including 3D volume (see Supplementary material).

### Exploratory model of GluCEST and cognitive screening measures

**Figure 4:**
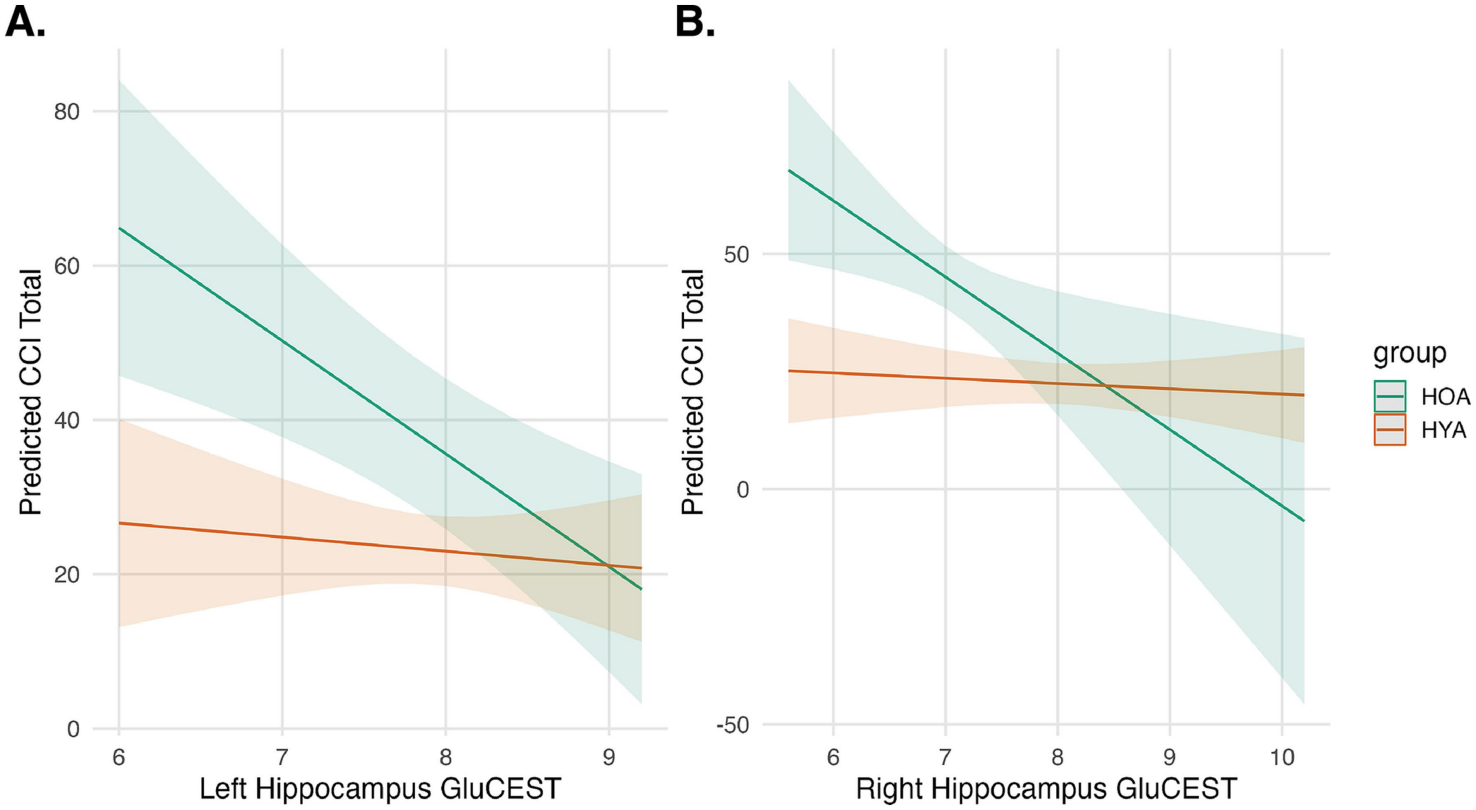
Hippocampal GluCEST and cognitive complaints. Predicted cognitive complaints (CCI Total) are shown as a function of Left **(A)** and Right **(B)** Hippocampal GluCEST for HOA (green) and HYA (orange). Shaded regions represent 95% confidence intervals. The interaction model showed an effect for group and a trend-level effect for GluCEST, indicating that younger age group and higher GluCEST are associated with fewer cognitive complaints.

MoCA scores were similar in HOA and HYA groups (Table 1). CCI scores were higher in HOA as compared to HYA (Table 1). An exploratory linear model of MoCA performance on GluCEST showed no main effects or interactions. The same model was also applied using CCI. This model showed a significant main effect for age group (*F*(1,56) = 42, p_FDR_ < 0.0001) and an effect GluCEST (F(1,56) = 4, *p* = 0.049) that did not survive correction for multiple comparisons (Figure 4). There was no main effect for hemisphere or any significant interactions.

## Discussion

The current study leveraged 7T GluCEST MRI to investigate hippocampal glutamate levels in healthy younger and older adults and revealed several significant findings. First, there was a significant interaction effect between age group and hemisphere, indicating that the relationship between GluCEST levels and hemisphere varies by age group. While GluCEST in the left hemisphere was higher than the right across all participants, this asymmetry was accentuated in older adults. A follow-up comparison of hippocampal GluCEST levels confirmed that only right hemisphere levels were lower in older adults compared to younger adults, and individual asymmetry measurements confirmed that older adults exhibited greater leftward asymmetry in GluCEST levels than younger adults. Sensitivity analysis confirmed that this interaction effect was attributable to differences in hippocampal volume. Finally, higher left and right GluCEST levels were correlated with lower subjective ratings of cognitive complaints but were not associated with performance on the MoCA.

Glutamate levels and signaling in the hippocampus undergo substantial changes with age, which likely influence cognitive function and brain health (Roalf et al., 2020). Aging is associated with changes in the expression and function of NMDA and AMPA receptors, leading to deficits in long-term potentiation (LTP), a process vital for memory formation (Kumar et al., 2019; Ménard and Quirion, 2012; Rozycka et al., 2019). In addition, aging and age-related disorders are often accompanied by increased neuroinflammation and oxidative stress, which can affect glutamate metabolism and signaling (Song et al., 2021). Inflammatory processes can lead to the dysregulation of glutamate transporters and receptors, exacerbating excitotoxic damage (Armada-Moreira et al., 2020).

*In vivo* Glu levels, as measured using ^1^HMRS, generally decline with age in various brain regions, including the hippocampus (Roalf et al., 2020). We found an age-related reduction in the right hippocampus. This approach expands upon previous ^1^HMRS studies indicating age-related Glu changes: GluCEST allowed for direct comparison of Glu across hemispheres and conferred improved sensitivity (Cai et al., 2012) and spatial resolution, so the signal was not contaminated by non-hippocampal tissue. Three previous ^1^HMRS studies conducted at 3T reported lower Glu or Glx with older age, but data were only collected in the left hippocampus (Hädel et al., 2013; Schubert et al., 2004) or right hippocampus (Huang et al., 2017), preventing systematic comparison between hemispheres. Our study contributes to the extant literature by enabling hemispheric comparison of Glu across a broad age range, leveraging high-precision GluCEST imaging in a robust sample size at 7T. Recent advancements in magnetic resonance spectroscopic imaging (MRSI) have also shown promise in generating high-resolution images (Weng et al., 2024) using spectroscopy techniques. MRSI has been used to characterize a decline in Glx (Glu + Gln) in the medial temporal lobe (Mahmoudi et al., 2023) and thalamus (Maghsudi et al., 2020) of healthy older adults. However, MRSI still faces limitations in resolution and acquisition time compared to GluCEST imaging, which achieves an unparalleled 1×1 mm in-plane resolution. Additionally, the overlap between Glu and Gln spectra remains a significant challenge in MRSI (Kanagasabai et al., 2024). Thus, GluCEST has the potential to provide unique, novel insights into glutamatergic changes associated with aging and age-related diseases. Future studies combining GluCEST and spectroscopy methods would leverage the strengths of both approaches.

Differences in Glu levels in the left and right hippocampus may reflect lateralized functional specialization (Shinohara et al., 2008). Translational research has shown lateralization of Glu functioning within the hippocampus (El-Gaby et al., 2015; Shinohara and Hirase, 2009; Shipton et al., 2014), but *in vivo* studies of Glu-related hippocampal asymmetry have been limited due to methodological constraints. Age-related Glu asymmetry may have important implications for understanding the potential age-related loss of lateralized functions of the hippocampus. For example, the left hippocampus, which had similar GluCEST levels in young and older groups, is more involved in verbal memory and language-related processes–functions that tend to be preserved in aging (Burgess et al., 2002; Ezzati et al., 2016). Conversely, the right hippocampus, which had lower GluCEST in older adults, is more associated with spatial memory and navigation–functions more commonly impaired with aging (Bohbot et al., 1998; Burgess et al., 2002; Ezzati et al., 2016). More broadly, age-related Glu changes in the hippocampus may align with general theories of aging such as the hemispheric asymmetry reduction in old adults (HAROLD) (Cabeza, 2002), which proposes that aging is characterized by a loss of cortical hemispheric specialization. If distinct properties of the left and right hippocampi contribute to hemispheric specialization, then asymmetrical changes in glutamatergic functioning could contribute to reduced hemispheric specialization and cognitive decline. Neurometabolic asymmetry may be a sign of incipient age-related disorders, such as mild cognitive impairment (Minkova et al., 2017), or reflect underlying compensatory mechanisms.

Few studies have measured the relationship between brain Glu and cognitive changes in HOA (Simmonite et al., 2019; Zahr et al., 2013; Zahr et al., 2008); however, those that have indicate that lower Glu is associated with poorer performance. We did not observe a direct relationship between GluCEST level and cognitive performance, as our primary measurement of cognition (MoCA) only provides a general index of functioning. Future studies should incorporate a more comprehensive assessment of specific cognitive domains. However, our exploratory analysis using the CCI suggested that lower bilateral GluCEST levels in older adults are related to increased subjective cognitive complaints, an indicator of cognitive decline in healthy older adults (Wang et al., 2006). Mechanistically, an age-related reduction in glutamate could affect the excitatory neurotransmission and synaptic plasticity essential for learning and memory.

The present findings are intriguing in light of a recent large-scale study showing accelerated volume loss in the left hippocampus compared to the right in older adults (Nobis et al., 2019). If this atrophy process is glutamate-driven, one would have expected lower GluCEST in the left hippocampus in older adults. However, cognitive function, spatial memory, and navigation–typically associated with the right hippocampus–decline precipitously after age 60 (Alexander et al., 2012). It is possible that glutamate loss in the right hippocampus is distinct from a pure atrophy process and contributes more directly to age-related functional decline. Alternatively, lower GluCEST in the right hippocampus may be protective against volume loss, perhaps through mitigation of cytotoxic damage. Longitudinal investigation incorporating precision imaging and behavioral phenotyping is warranted to uncover the interplay between hippocampal volume loss, glutamatergic changes, and cognitive decline.

Understanding glutamate changes in the hippocampus may offer valuable insight into biological mechanisms of aging and age-related disorders. In response to declining Glu levels and receptor functionality, the aging brain may engage compensatory mechanisms, such as upregulation of remaining receptors or increased reliance on alternative neural circuits (Morcom and Johnson, 2015), to maintain cognitive functions. Many neurodegenerative diseases may share glutamate-induced excitotoxicity as a common pathogenic pathway (Dong et al., 2009). For example, altered brain Glu in Alzheimer’s Disease (AD) (Graff-Radford and Kantarci, 2013) could lead to or result from excitotoxic damage; a growing body of evidence suggests that the mechanism through which Aβ incites cognitive decline is by modulation of the glutamatergic system (Findley et al., 2019). Indeed, patients with probable AD show elevated cerebrospinal fluid levels of glutamate and glutamine (Madeira et al., 2018). Moreover, the use of NMDA receptor antagonists, such as memantine and amantadine, has been shown to improve cognition, daily functioning, and certain neuropsychiatric symptoms in AD patients (Herrmann et al., 2011). However, the limited clinical efficacy of these agents may stem from late intervention when neuronal disruption is too far advanced (Folch et al., 2018). Elucidating glutamate changes in healthy aging could thus provide vital insights that may guide the development of more timely and targeted therapeutic interventions for age-related neurological disorders.

While this study offers an unprecedented look at *in vivo* hippocampal glutamate levels, limitations should be considered. First, the small sample size limited statistical power, particularly for the exploratory GluCEST-behavior analyses. Logistical constraints imposed by COVID-19-related campus regulations resulted in only 10 older adults completing the CCI during their visit. Additionally, because we focused on healthy aging, participants diagnosed with cognitive decline were excluded, which may have reduced phenotypic variability within the older adult group. The sample size was also insufficient to analyze men and women separately, but sensitivity results indicate that both older men and women show lower GluCEST and greater GluCEST asymmetry than their younger counterparts. Biological sex is an important consideration for future studies, as it has been linked to age-related cognitive changes (Yagi and Galea, 2019) and differential gene expression in the hippocampus (Achiro et al., 2024; Guebel and Torres, 2016). While we addressed biological sex using covariates and sensitivity analyses, future research should further explore these effects in men and women more explicitly. Despite efforts to recruit a diverse cohort, Asian populations were underrepresented in the HOA group. The impact of this discrepancy remains unclear, but future studies should aim to recruit larger, more representative samples to better understand potential demographic influences on hippocampal Glu. GluCEST contrast also has inherent limitations. First, the 2D FOV of the GluCEST acquisition constrained analysis to a portion of the hippocampus, potentially contributing to some of the asymmetry effects described. However, the measured signal from GluCEST is localized exclusively to hippocampal volumes, offering a substantial improvement in spatial specificity compared to traditional ^1^HMRS techniques. Furthermore, sensitivity analyses incorporating 3D volume supported our main findings. The 2D FOV was optimized for hippocampal imaging and did not capture sufficient data from other regions, such as the amygdala, to include a control region. However, GluCEST contrast is an absolute measure; as with ^1^HMRS, comparison to a control region is not strictly necessary (Zaiss and Mennecke, 2023). Future GluCEST studies should leverage new advances in 3D GluCEST acquisition to replicate the present findings across the whole hippocampus and explore glutamatergic trends across other subcortical regions (Jacobs et al., 2022). At least 70% of the CEST effect is directly attributable to glutamate, with no contamination from glutamine. However, up to 30% of the signal can come from other macromolecules (e.g., creatine) (Cai et al., 2012). GluCEST contrast is also sensitive to tissue pH (Cai et al., 2012; Kogan et al., 2013). To our knowledge, age- or laterality-related pH differences in the hippocampus have not been documented, but future research may benefit from incorporating sequences that measure pH (Maintz et al., 2002). Lastly, unlike ^1^HMRS, GluCEST does not provide information about other neurochemicals (e.g., N-acetyl aspartate, choline, GABA, etc.). Due to limited scan time, we were unable to collect spectroscopy data in the present study. Future studies may consider acquiring complementary GluCEST and ^1^HMRS or MRSI data to capitalize on the enhanced coverage and spatial resolution of GluCEST and the localized measurement of multiple metabolites enabled by ^1^HMRS.

In summary, by harnessing the ultra-high field GluCEST imaging, we identified an asymmetrical reduction in brain glutamate in healthy older adults that was related to subjective cognitive complaints, implicating hippocampal glutamatergic deficiencies in both aging and age-related functioning. Future longitudinal studies are warranted to parse relationships between brain Glu, hippocampal function, and progression to neurodegenerative disorders. More broadly, this study demonstrates the utility of *in vivo* neurochemical imaging, highlighting its potential to identify modifiable targets for understanding the dynamics of Glu change with age, which may pave the way for developing interventions aimed at mitigating age-related cognitive decline and preventing neurodegenerative diseases.

## Data Availability

The raw data supporting the conclusions of this article will be made available by the authors upon request.
