# Supplementary material for: Mapping hippocampal glutamate in healthy aging with in vivo glutamate-weighted CEST (GluCEST) imaging
Source: Front Aging Neurosci. 2025 Jan 24;16:1535158. doi: 10.3389/fnagi.2024.1535158 (PMC11802501; doi:10.3389/fnagi.2024.1535158)
Supplement: Supplementary file 1 [file Data_Sheet_1.docx]

***Supplemental Material***

**Methods:**

**3T Structural MRI Acquisition**

A subset of participants (n=26 HOA, n=14 HYA) also had 3T T1w 3D volumetric data available. 3T high resolution MRI was collected as part of other on-going projects. The sequence was a sagittal MPRAGE with voxel resolution(s) of 0.8-1.0 mm × 0.8-1.0 mm and slice thickness of 0.8-1.0 mm with ranges of TE = 2400–2900 ms, TR = 2.4–2.5 ms, and TI = 1060–1070 msec based on scanner bore size and gradient strengths. The dimensions of each scan were 176-208 × 256-300 voxels per slice and 256-320 slices. A Siemens phased-array head and neck coil was used with 7 head coil elements (HC1-7) and 1 neck coil element (NC1) active.

**3T MRI Data Processing**

Volumetric analyses were completed using the standard ‘recon-all’ pipeline in Freesurfer (Fischl et al., 2002).

**Table S1: Scanning Quality**

|  | **HOA** | **HYA** | **P-value** |
| --- | --- | --- | --- |
|  | **(N=27)** | **(N=22)** |  |
| **Motion mean** | |  |  |
| Mean (SD) | 0.176 (0.110) | 0.215 (0.231) | 0.469 |
| Median [Min, Max] | 0.169 [0.0520, 0.486] | 0.136 [0.0419, 1.11] | |
| **Motion max** | |  | |
| Mean (SD) | 0.322 (0.166) | 0.449 (0.428) | 0.201 |
| Median [Min, Max] | 0.291 [0.115, 0.841] | 0.254 [0.108, 1.72] | |

Scanning quality during the 7T GluCEST acquisition was high in both age groups. Mean and maximum relative motion did not differ between age groups.

**Table S2: 3T Volume Data**

| **HOA** | | **HYA** | **P-value** |
| --- | --- | --- | --- |
| **(N=26)** | | **(N=14)** |  |
| **Left Hippocampus**  **3D volume (mm^3^)** | |  |  |
| Mean (SD) | 3640 (380) | 4230 (470) | <0.001 |
| Median [Min, Max] | 3630 [2980, 4210] | 4130 [3640, 5360] | |
| Missing | 1 (3.7%) | 8 (36.4%) |  |
| **Right Hippocampus**  **3D volume (mm^3^)** | |  |  |
| Mean (SD) | 3800 (468) | 4360 (497) | 0.00195 |
| Median [Min, Max] | 3780 [2670, 4550] | 4360 [3710, 5460] | |
| Missing | 1 (3.7%) | 8 (36.4%) |  |
| **ETIV* (mm^3^)** |  |  |  |
| Mean (SD) | 1.59e6 (1.72e5) | 1.62e6 (2.64e5) | 0.653 |
| Median [Min, Max] | 1.59e6 [1.2e6, 1.89e6] | 1.68e6 [9.6e6, 1.86e6] | |

*ETIV=Estimated total intracranial volume

3T hippocampal volume was significantly lower in both left and right hemisphere in HOA as compared to HYA. ETIV did not differ significantly between groups.

**Results:**

*Sensitivity analysis for motion:*

There were no significant associations between head motion parameters and GluCEST.

*Sensitivity analysis for biological sex:*

T tests were used to compare the asymmetry effect in males and females separately.

- **HOA vs. HYA Asymmetry in Females**: t(28.8)=3.1,p=0.0045
- **HOA vs. HYA Asymmetry in Males**: t(11.3)=1.9,p=0.08.

*Sensitivity analysis for volume asymmetry:*

Asymmetry analysis of 3D hippocampal volume showed that volumetric asymmetry was not associated with age-group or gender.

*Sensitivity analysis for mixed model analysis in Figure 2A and B:*

As a sensitivity analysis, the mixed model from Figure 2 was also repeated using 3D volume, instead of 2D volume, as a covariate. The age-group-hemisphere interaction remained significant (F(1, 47.1) = 10.62, p=0.0017). There was also an effect for 3D hippocampal volume (F(1,47.1) = 8.27, p=0.005), but this variable was highly colinear with the group variable, limiting interpretability.

*Sensitivity analysis for asymmetry analysis in Figure 2C:*

The GluCEST asymmetry analysis was repeated using 3D hippocampal volume as a covariate. The difference in GluCEST asymmetry between the HOA and HYA groups remained significant (F(1,36)=8.8, p<0.005) and there was no significant effect for gender or 3D volume asymmetry.
